# Gene expression analysis of bone metastasis and circulating tumor cells from metastatic castrate-resistant prostate cancer patients

**DOI:** 10.1186/s12967-016-0829-5

**Published:** 2016-03-15

**Authors:** Won Jin Cho, Daniel S. M. Oliveira, Abdo J. Najy, Leandro E. Mainetti, Hussein D. Aoun, Michael L. Cher, Elisabeth Heath, Hyeong-Reh C. Kim, R. Daniel Bonfil

**Affiliations:** Department of Urology, Wayne State University School of Medicine and Karmanos Cancer Institute, 540 E. Canfield, Scott Hall # 9105, Detroit, MI 4820 USA; Department of Pathology, Wayne State University School of Medicine and Karmanos Cancer Institute, Detroit, MI USA; Department of Radiology, Wayne State University School of Medicine and Karmanos Cancer Institute, Detroit, MI USA; Department of Oncology, Wayne State University School of Medicine and Karmanos Cancer Institute, Detroit, MI USA

**Keywords:** Circulating tumor cells, Laser capture microdissection, Prostate cancer, Bone metastasis, Gene expression

## Abstract

**Background:**

Characterization of genes linked to bone metastasis is critical for identification of novel prognostic or predictive biomarkers and potential therapeutic targets in metastatic castrate-resistant prostate cancer (mCRPC). Although bone marrow core biopsies (BMBx) can be obtained for gene profiling, the procedure itself is invasive and uncommon practice in mCRPC patients. Conversely, circulating tumor cells (CTCs), which are likely to stem from bone metastases, can be isolated from blood. The goals of this exploratory study were to establish a sensitive methodology to analyze gene expression in BMBx and CTCs, and to determine whether the presence or absence of detectable gene expression is concordant in matching samples from mCRPC patients.

**Methods:**

The CellSearch^®^ platform was used to enrich and enumerate CTCs. Low numbers of PC3 prostate cancer (PCa) cells were spiked into normal blood to assess cell recovery rate. RNA extracted from recovered PC3 cells was amplified using an Eberwine-based procedure to obtain antisense mRNA (aRNA), and assess the linearity of the RNA amplification method. In this pilot study, RNAs extracted from CTCs and PCa cells microdissected from formalin-fixed paraffin-embedded BMBx, were amplified to obtain aRNA and assess the expression of eight genes functionally relevant to PCa bone metastasis using RT-PCR.

**Results:**

RNAs were successfully extracted from as few as 1–5 PCa cells in blood samples. The relative expression levels of reference genes were maintained after RNA amplification. The integrity of the amplified RNA was also demonstrated by RT-PCR analysis using primer sets that target the 5′-end, middle, and 3′-end of reference mRNA. We found that in 21 out of 28 comparisons, the presence or absence of detectable gene expression in CTCs and PCa cells microdissected from single bone lesions of the same patients was concordant.

**Conclusions:**

This exploratory analysis suggests that aRNA amplification through in vitro transcription may be useful as a method to detect gene expression in small numbers of CTCs and tumor cells microdissected from bone metastatic lesions. In some cases, gene expression in CTCs and BMBxs was not concordant, raising questions about using CTC gene expression to make clinical decisions.

**Electronic supplementary material:**

The online version of this article (doi:10.1186/s12967-016-0829-5) contains supplementary material, which is available to authorized users.

## Background

More than 80 % of patients with metastatic castrate-resistant prostate cancer (mCRPC) present skeletal metastases, which invariably lead to an incurable disease for which we only have treatments that become palliative in nature [1, 2]. Thus, the identification of gene signatures pertaining to prostate cancer (PCa) bone metastasis is fundamental to the development of novel therapeutic targets and/or the identification of prognostic or predictive biomarkers.

The vast majority of gene profile analyses using biopsies from PCa patients have been performed in primary tumors or metastatic lesions other than bone [3–5], which might not provide clues about gene transcriptional changes occurring in skeletal metastasis. Although gene analysis of bone marrow core biopsies (BMBxs) would be of importance to identify the genetic make-up of PCa cells that colonize and interact with the bone microenvironment, BMBxs are not routinely performed in mCRPC patients due to the time-consuming and invasive nature of the procedure. Furthermore, in most cases, the combination of imaging studies and clinical information has proven to provide diagnostic accuracy for the assessment of suspected bone metastases [6]. In contrast, circulating tumor cells (CTCs) can be obtained repeatedly and non-invasively through routine blood draws, and isolated through different techniques utilizing cell-surface antigens, or other physical, and/or biological properties of cancer cells [7–9].

CTCs are rare cancer cells transported through the peripheral circulation. In localized, non-metastatic cancers, CTCs are thought to emanate from primary tumors. CTCs may also be released from metastatic lesions and found in peripheral blood of patients with advanced cancer, thus providing an opportunity for “liquid biopsy” that may offer information on the evolution of the disease during treatment [9, 10]. The assessment of CTC numbers using the CellSearch^®^ platform has been validated as a tool of clinical utility to monitor treatment response and predict survival in mCRPC patients [11].

Previous studies by others have demonstrated clearly the capacity of gene expression analyses of RNA obtained in large amounts from bone metastases derived from rapid autopsies of PCa patients [12, 13]. Here, we wished to determine the feasibility of gene expression analysis of both CTCs and BMBxs in living mCRPC patients with bone scan evidence of skeletal metastasis. Since CTCs in mCRPC patients are likely to derive from bone metastatic deposits, we also aimed to explore similarities/differences in gene expression in both CTCs and BMBxs obtained from the same patients.

## Methods

### Cell culture

PC3 and LNCaP PCa cells were obtained from ATCC (Manassas, VA) and provided by Dr. Leland Chung (Cedars-Sinai Medical Center, Los Angeles, CA), respectively. Both cell lines were grown in RPMI-1640 with 10 % heat-inactivated fetal bovine serum (FBS). Authentication of the human cell lines used here was verified through short tandem repeat profiling by the Research Technology Support Facility of Michigan State University.

### Cell spike-in experiments

For validation studies, 80 % confluent PC3 and LNCaP monolayers were trypsinized, and diluted in complete culture medium to adjust their cell density to 50 cells/mL. Cell suspensions (100 μL) were plated into the wells of 96-well cell culture plates. Twenty-four hours later, cells in all wells were counted under the microscope, and those with exactly five cells were trypsinized and added to 7.5 mL of blood from healthy volunteer donors drawn into CellSave^®^ preservative tubes (Janssen Diagnostics, LLC). CellSave^®^ tubes containing blood spiked with cultured PCa cells, as well as matched non-spiked blood samples serving as negative controls, were processed in triplicate using the semiautomated CellSearch^®^ Circulating Epithelial Cell Kit (Janssen) in the CellTracks^®^ Autoprep^®^ system (Janssen) for cell enumeration at the Biobanking and Correlative Sciences Core at Karmanos Cancer Institute. Briefly, epithelial cells present in peripheral blood were magnetically captured with a ferrofluid-coupled antibody targeting the epithelial cell adhesion molecule (EpCAM), then immunostained with allophycocyanin (APC)-labeled antibodies to the leukocyte marker CD45, phycoerythrin (PE)-labeled antibodies to epithelial markers cytokeratins (CKs) 8, 18, and 19, and stained with the nuclear stain 4,2-diamidino-2-phenylindole-dihydrochloride (DAPI). The sample was transferred automatically to a cartridge in a MagNest, and finally scanned with the semi-automated fluorescence optical system Cell-Tracks Analyzer II^®^ (Janssen). Objects preselected and displayed by the system in a gallery were defined as CTCs and counted by a trained operator if they were round to oval in shape, 4 µm in size or larger, positive for the epithelial marker (CK-PE) and nuclear stain (DAPI) with at least 50 % overlap between the CK-PE-positive cytoplasm and the nucleus, and negative for the leukocyte marker (CD45-APC). In addition, 5 PC3 or LNCaP cells were spiked into 7.5 mL of blood from healthy volunteer donors drawn into fixative-free K3EDTA tubes (BD Vacutainer^®^, Becton–Dickinson) (three experiments performed by three different investigators), to validate the sensitivity of the RNA amplification method used by us (see description below). To this end, the spiked blood was immediately processed using the CellSearch^®^ Profile Kit (Janssen) and the CellTracks^®^ Autoprep^®^ system, and then the tube employed containing a final volume of 900 µL CTC enriched sample was placed in a DynaMag™-15 magnet (Invitrogen) for 10 min. After carefully aspirating the liquid without disturbing the ferrofluid bead pellet concentrated on the tube wall, 1 mL of TRIzol^®^ (Invitrogen) was added, and the tube vortexed to lyse cells and inactivate nucleases. The lysate was transferred to a 1.5 mL RNase-free Eppendorf tube, and stored at −80 °C until RNA isolation and antisense RNA (aRNA) amplification was performed.

### Patient enrollment and eligibility

Written informed consent was obtained from 24 mCRPC patients enrolled to participate in the human protocol # 2011-060 (Principal Investigator: Dr. Michael L. Cher), and approved by Karmanos Cancer Institute and Wayne State University Institutional Review Board. Study inclusion criteria were: (a) prior diagnosis of mCRPC characterized by rising PSA level or clinical disease progression despite a castrate level of serum testosterone; (b) clinical decision to start a new anti-cancer systemic therapy, and no treatment with any investigational drug within 2 weeks prior to blood draw/tissue biopsy proposed herein; (c) metastatic deposit visible on an imaging study obtained within 8 weeks prior to initiation of new systemic therapy; (d) radiological evidence of accessibility to a metastatic deposit in bone by computed tomography (CT); (e) performance status of 0–2 by ECOG/Zubrod criteria; (f) Absolute neutrophil count ≥1500 mm^3^, hemoglobin ≥9.0 g/dL, platelets ≥100,000/mm^3^; (g) Prothrombin Time and Partial Thromboplastin Time should be < institutional upper limit of normal; (h) presence of one or more CTCs per 7.5 mL, assessed using the Veridex CellSearch^®^ Profile Kit assay, as explained above. The characteristics of the patients enrolled in this study are described in Table 1.

**Table 1:** Demographic and clinical parameters of evaluable mCRPC patients

Variable	Categories	Value
Age	Median	69
	Range	58–88
Race	White	10 (67)
	Black	5 (33)
	Hispanic	0 (0)
	Asian	0 (0)
Gleason score^a^		5 (33)
	8–10	8 (53.5)
	Not available	2 (13.5)
PSA (ng/mL)	Median	70.8
	Range	1.9–1299
AP (U/L)	Median	437.5
	Range	65–1224
Metastases	Bone only	11 (73)
	Bone and soft tissue	2 (13.5)
	Bone and unknown^b^	2 (13.5)
CTC/7.5 mL	Median	20
	Range	1–834

Data between parentheses are percentage

*AP* alkaline phosphatase; *CTC* circulating tumor cell; *PSA* prostate-specific antigen

^a^ At time of enrollment. Not available for three patients due to unavailability of pathological results

^b^ Patients with suspicious lesions under investigation at the time of study enrollment

### Blood sample processing

Blood from each patient was initially collected in CellSave^®^ preservative tubes and processed for CTC enumeration as described above. Patients who were positive for CTCs were subjected to a new blood draw into K3EDTA anticoagulant tubes used in the CellSearch^®^ Profile Kit, for CTC enrichment and RNA extraction as described above. The TRIzol^®^ lysate obtained was transferred to a 1.5 mL RNase-free Eppendorf tube, and stored at -80 °C until needed.

### Procurement and processing of tumor tissue from bone metastases

On the same day blood was drawn for CTC enrichment and RNA extraction, patients then underwent CT-guided BMBx using a battery-powered drill and biopsy needle set. Four to six BMBxs were obtained from the iliac bone of each patient. One of the cores was placed in an RNase-free Eppendorf tube and flash frozen and transported in liquid nitrogen for storage at −80 °C until processing. The remaining BMBxs were fixed for 24 h in 4 % paraformaldehyde in diethylpyrocarbonate (DEPC)-treated PBS, and decalcified in 10 % EDTA in autoclaved DEPC-treated water, pH 7.0, with agitation at room temperature for 3 days. After decalcification, each BMBx was progressively dehydrated with increasing concentrations of ethanol, and immediately paraffin-embedded using Precision Cut Paraffin (Thermo Scientific). All aqueous solutions were prepared using DEPC-treated water.

### Laser capture microdissection

Two adjacent 5-µm sections were obtained from the formalin-fixed paraffin-embedded (FFPE) BMBxs, using at all times RNase-free technique. Immunohistochemistry (IHC) for cytokeratin was performed on one of the slides for identification of PCa cells, as previously described [14, 15], as PCa cells are the only cytokeratin-positive cells expected to be found within the BMBx. The adjacent tissue section was mounted onto an RNase-free polyethylene naphthalate (PEN) membrane glass slide (Arcturus), which was previously sprayed with RNase AWAY™ (Thermo Scientific), washed twice with DEPC-treated, nuclease-free water (Fisher Scientific), and then exposed to UV for 30 min under a laminar flow hood to render the PEN membrane more hydrophilic and improve adherence of the specimen. After air drying for about 2 h under a laminar flow hood, the section mounted on the slide was deparaffinized, hydrated with decreasing graded alcohols made with DEPC-treated water, and rapidly stained with Harris hematoxylin and alcoholic Eosin Y solution (H&E), washed twice with 100 % ethanol and xylene, then held in xylene until initiation of the laser capture microdissection (LCM) session. The H&E-stained slide was air dried and loaded onto the ArcturusXT™ LCM System. Metastatic lesions identified in the FFPE sample were excised using both the infrared (IR) and ultraviolet (UV) microdissection lasers, and collected on individual CapSure^®^ Macro LCM Caps (Arcturus). When needed, the adjacent section immunostained for cytokeratin was used for guidance during the LCM session to identify epithelial (PCa) cells within the bone marrow. Tumor material was deemed to be inadequate for LCM and RNA collection if the area identified as tumor was smaller than 9000 µm^2^, with 150 PCa cells on average. To collect total RNA from the microdissected tissue, the CapSure^®^ Macro LCM Cap containing the captured material was immediately placed into a 0.5 mL RNase-free microcentrifuge tube filled with 50 µL of TRIzol^®^ reagent, vortexed for 5 min to lyse the cells, and the lysate obtained stored at −80 °C until needed.

### RNA isolation and amplification of small amounts of RNA

Cultured PCa cells and CTCs were lysed with TRIzol^®^ reagent, as described above. Flash frozen BMBxs were placed in pre-chilled microtubes containing 2.8 mm ceramic beads and TRIzol^®^ reagent using a Precellys^®^24 homogenizer (Peqlab LLC), 1 cycle × 30 s at 6000 rpm at 4 °C, similarly as described by others [16]. High-quality total RNA (DNA-free) was purified from cultured cells, CTCs, LCM, and frozen BMBxs lysed with TRIzol^®^ using Direct-zol™ RNA MiniPrep (Zymo Research), as per manufacturer’s instructions. For gene analysis of CTCs enriched from patients’ blood, spiked-in cultured cells, or tumor cells metastatic to bone recovered by LCM, RNAs were amplified using an antisense mRNA (aRNA) amplification system based on the Eberwine’s procedure [17], using the MessageAmp™ II aRNA Amplification Kit (Invitrogen). The protocol was performed as recommended by the manufacturer, except for the replacement of ArrayScript™ RT by Superscript^®^ VILO™ Master Mix (Invitrogen) and the replacement of 10 × First Strand buffer from MessageAmp™ II aRNA Amplification Kit (Invitrogen) by 5 ×  First Strand buffer from Superscript^®^ II Reverse Transcriptase (Invitrogen). This was done to increase the reverse transcription (RT) of total RNA into a first strand of complimentary DNA (cDNA). SuperScript^®^ VILO™ Master Mix contains a recombinant ribonuclease inhibitor and SuperScript^®^ III RT, which is among the best performing reverse transcriptases in terms of reproducibility and sensitivity for low copy RNA levels [18]. After RNase treatment, a second strand cDNA is generated by DNA polymerase. The resulting double-stranded cDNA was then used as a template for T7-RNA polymerase for in vitro transcription (IVT) into aRNA (also known as complimentary RNA, cRNA) and amplification. The procedure was repeated in a second round of amplification when additional aRNA yield was needed. After IVT, double-stranded cDNAs were removed by treatment with DNase I, and the amplified RNA was purified.

Concentrations of total RNA and amplified aRNA obtained from cells spiked in blood, as well as from amplified aRNA obtained from CTCs and flash frozen or FFPE BMBxs, were quantified by absorbance measurements using an Epoch™ Microplate Spectrophotometer for micro-volume analysis (BioTek). Purity of total RNA and amplified aRNA was assessed through the ratio of the absorbance of the samples at 260 and 280 nm (A_260_/A_280_).

### Assessment of amplified aRNA integrity

Integrity of mRNA is usually assessed by the ratio of the 28S:18S rRNA species shown in denaturing agarose gel electrophoresis, based on the assumption that the quality of rRNA (more than 80 % of total RNA) reflects that of underlying mRNA. However, this approach cannot be used to assess the integrity of amplified aRNA, which does not contain rRNA and derives from minute amounts of total RNA. Therefore, we assessed the integrity of aRNA with RT-PCR (see below) using two sets of primers to probe different positions (5′ end, middle, and 3′ end regions) of *EpCAM* and *GAPDH* transcripts, by verifying the presence of each respective amplicon on agarose gels (Fig. 2a), following a strategy similar to that described by Nolan [19].

### Gene expression analysis

Total RNA or amplified aRNA was reverse transcribed into cDNA using iScript™ cDNA synthesis kit (Bio-Rad) according to manufacturer’s protocol. For RT-PCR, resultant cDNAs were used as a template in a PCR reaction using DreamTaq DNA polymerase (Life Technologies). Forward and reverse primers used are listed in Table 2. The following amplification conditions were used: an initial denaturation at 95 °C for 3 min, followed by 35–40 cycles (except for GAPDH, 25 cycles) of 95 °C for 30 s, 52–55 °C for 30 s and 72 °C for 1 min, followed by a final extension 72 °C for 5 min. PCR products were resolved on a 2 % agarose gel and visualized by ethidium bromide staining. DNA bands were visualized using a ChemiDoc XRS gel documentation system (Bio-Rad).

**Table 2:** Primer pairs used for RT-PCR and RT-qPCR studies

Gene ID	Accession number	Oligonucleotides (5′ →3′)	Amplicon size (bp)
*BMP7*	NM 001719	FWD: TACGCCGCCTACTACTGTGA REV: CCGGACCACCATGTTTCTGTA	219
*CD45*	NM 002838	FWD: AGCACCTACCCTGCTCAGAA REV: TTCAGCCTGTTCCTTTGCTT	159
5′-*EpCAM*	NM 002354	FWD: CAGGTCCTCGCGTTCGGG REV: CAGTCAGGATCATAAAGCCCATCA	284
Middle *EpCAM*	NM 002354	FWD: AATGGACCTGACAGTAAATGG REV: ATCTCAGCCTTCTCATAC TT	216
3′-*EpCAM*	NM 002354	FWD: TGGGGAACAACTGGATCTGG REV: GTTCCCTATGCATCTCACCCA	227
5′-*GAPDH*	NM 002046	FWD: GGAAGGTGAAGGTCGGAGTC REV: CTCGCTCCTGGAAGATGGTG	237
Middle *GAPDH*	NM 002046	FWD: GAGAAGGCTGGGGCTCATTT REV: AGTGATGGCATGGACTGTGG	231
3′-*GAPDH*	NM 002046	FWD: AAGGTCATCCCTGAGCTGAA REV: TGACAAAGTGGTCGTTGAGG	271
*IL6*	NM 000600	FWD: AATGAGGAGACTTGCCTGGTG REV: GCTGCGCAGAATGAGATGAG	273
*MMP14*	NM 004995	FWD: AGTCTCCCAGAGGGTCATTCA REV: GGTCCCATGGCGTCTGAAG	320
*SNAI 2*	NM 003068	FWD: CTTTTTCTTGCCCTCACTGC REV: ACAGCAGCCAGATTCCTCAT	161
*t*-*ERG*	FJ423744	FWD: TMPRSS2_E1-TAGGCGCGAGCTAAGCAG REV: EGR_E4-GTCCATAGTCGCTGGAGGAG	184
*Vimentin*	NM 003380	FWD: GAGAACTTTGCCGTTGAAGC REV: TCCAGCAGCTTCCTGTAGGT	170
*ZEB 1*	NM 030751	FWD: TGCACTGAGTGTGGAAAAGC REV: TGGTGATGCTGAAAGAGACG	237

*FWD* forward primer; *REV* reverse primer; *bp* base pairs; *t*-*ERG*, *TMPRSS2 exon 1 and ERG exon 4 (TMPRSS2*-*ERG) fusion product*

For reverse transcriptase quantitative real-time quantitative PCR (RT-qPCR), the Mastercycler RealPlex2 (Eppendorf) real-time PCR system and GoTaq qPCR Master Mix (Promega) were used. Thermal cycle parameters were as follows: initial activation at 95 °C for 2 min, 40 cycles of denaturation at 95 °C for 15 s, annealing 55 °C for 15 s, and extension at 72 °C for 30 s. The mean cycle threshold (Ct) for each gene was normalized to levels of the housekeeping gene *GAPDH* in the same sample. Relative fold changes in expression for each gene were calculated by the delta–delta-CT method [20].

As a proof of concept, we selected eight genes for analysis with RT-PCR in CTCs and LCM BMBxs based on their relevance to PCa bone metastasis: (a) *EpCAM*, which codes for a transmembrane epithelial glycoprotein [21] overexpressed in adenocarcinomas [22] and used in the CellSearch^®^ system to enrich CTCs via immunomagnetic separation; (b) *PSA* (prostate-specific antigen), a well know biomarker for PCa screening found to be positive in mCRPC patients [23]; (c) *BMP*-*7* (bone morphogenetic protein-7), a member of the transforming growth factor-beta (TGF-β) family that is usually expressed in osteoblastic bone metastases of PCa [24], and increased in CRPC patients [25]; (d) *MMP*-*14* (a.k.a. *MT1*-*MMP*), a membrane-tethered matrix metalloproteinase that we found to be expressed by PCa cells in skeletal metastases, and contribute to bone remodeling and intraosseous tumor growth [26]; (e) *TMPRSS2*-*ERG* gene rearrangement due to chromosomal translocations that fuse the androgen-regulated *TMPRSS2* promoter with the ETS family transcription factor *ERG*, which is expressed in about half of advanced PCas [27–29] and has been associated with an increased risk death in PCa patients [30–32]; (f) *IL*-*6* (interleukin-6), which is highly expressed by PCa cells with aggressive phenotype [33], and has been associated with resistance to chemotherapy in CRPC [34] and bone remodeling in PCa bone metastases [35, 36]; (g) *Vimentin*, an intermediate filament protein expressed in mesenchymal cells frequently used as a marker of epithelial to mesenchymal transition (EMT) [37]; and (h) *GAPDH*, a housekeeping gene used as a control.

### Statistical analysis

Data obtained using RT-qPCR are presented as mean values ± SD and analyzed using the ANOVA test, with p < 0.05 considered as statistically significant.

## Results

### Validation of linear amplification of RNA for gene analysis in small number of PCa cells

**Fig. 1:**
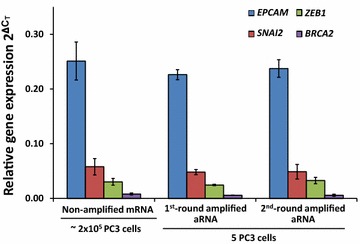
Comparison of gene transcript levels in total RNA and amplified aRNA. Transcriptional profiles assessed by RT-qPCR for genes with high (*EpCAM*), intermediate (*SNAI2, ZEB1*), and low (*BRCA2*) expression levels in total RNA from high number of cultured PC3 cells, and amplified aRNA from five PC3 cells spiked in blood after CellSearch^®^ Profile kit processing. Relative expression of genes was normalized to that of *GAPDH*, calculated by the delta–delta-CT method. No significant statistical differences in relative expression for each particular transcript were observed among non-amplified mRNA, one-round amplified aRNA, and two-round amplified aRNA, as per analysis of variance (ANOVA)

To mimic the CTC number threshold currently used to predict overall survival (OS) in men with mCRPC [11, 21, 38–40] and establish the method for RNA amplification from limited quantities of total RNA for gene expression, we spiked blood from healthy male volunteers with five PC3 or 5 LNCaP cells. Using the CellSearch^®^ Circulating Epithelial Cell Kit in the CellTracks^®^ Autoprep^®^ system, we found that the recovery rate of PC3 and LNCaP cells spiked into normal blood was 76.7 ± 6.2 % (n = 6) and 80.2 ± 3.0 % (n = 6) at the five and 50 cell level, respectively, confirming high efficiency of PCa cell recovery from blood. No epithelial cells were found in non-spiked matched blood used as negative controls. After we confirmed the cell recovery rate using the Circulating Epithelial Cell enrichment system, we then spiked five PC3 or LNCaP cells in new blood of healthy men drawn in K3EDTA tubes and processed using the CellSearch^®^ Profile Kit, used for RNA analysis. Minute amounts of total RNA extracted from the PC3 and LNCaP cells recovered through that CellSearch^®^ Profile Kit and the CellTracks^®^ Autoprep^®^ system were subjected to one and two round amplification using the MessageAmp™ II aRNA Amplification Kit. As shown in Table 3, this methodology was efficient enough to generate sufficient quantities of aRNA of good quality (A_260_/A_280_ ratio of 1.8–2.0) from a few PCa cells for gene expression analyses, such as RT-PCR, RT-qPCR, and cDNA microarrays if desired. To establish the nature of linear amplification, we compared relative expression levels in a set of genes with high, medium, or low expression levels, in unamplified RNA extracted from 2 × 10^5^ PC3 cells (~6–9 µg of total RNA, equivalent to ~180–270 pg of mRNA) and one- or two-round amplified aRNA derived from five PC3 cells (Table 3). The relative abundance of transcripts for the different genes assessed by RT-qPCR was maintained, confirming the linear amplification of RNA (Fig. 1).

**Table 3:** Yield of aRNA after one and two rounds of amplification of RNA obtained from five cultured prostate cancer cells

Cell line	Median (range) total RNA/5 cells (pg)^a^	Median (range) mRNA/5 cells (pg)^b^	Median (range) yield of aRNA/5 cells (ng)
PC3	194.3 (150–233)	5.83 (4.5–6.9)	495 (94.4–630) (1st round)
			4650 (1200–7200) (2nd round)
LNCaP	130.0 (105–155)	3.9 (3.2–4.7)	525 (310–680) (1st round)
			4950 (1500–6300) (2nd round)

^a^ Calculated based on measurement of total RNA from 10^6^ cells

^b^ Based on the assumption that 3 % of total RNA is mRNA

### Gene expression analysis in CTCs and BMBx from mCRPC patients with bone metastasis

**Fig. 2:**
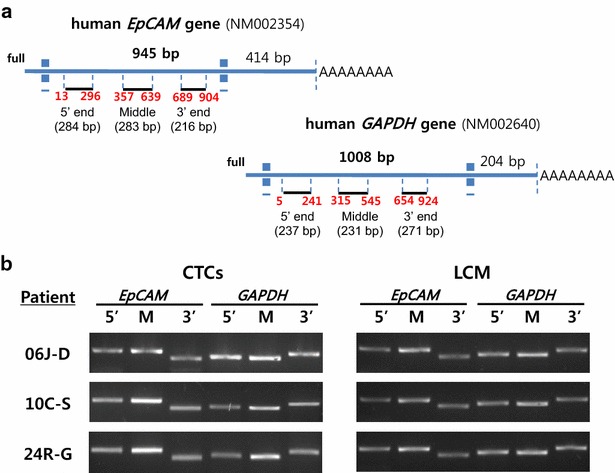
Quality of aRNA amplified from CTCs and LCM bone metastases from mCRPC patients. **a** Primers designed to amplify 5′-end, middle (M), and 3′-end fragments of *EpCAM* and *GAPDH* transcripts. **b** RT-PCR products obtained for amplified aRNA derived from CTCs and laser microdissected tumor cells metastatic to bone of three mCRPC patients, using the two sets of primers targeting different regions of *EpCAM* and *GAPDH*

To check the purity and quality of the aRNA amplified from RNA extracted from CTCs and PCa cells microdissected from BMBxs, we designed primers to target different regions of *EpCAM* and *GAPDH* transcripts, epithelium-specific and housekeeping genes, respectively (Fig. 2a). The primers targeting the 5′ end and the middle regions of the transcripts produced the expected size of the resultant PCR products as effectively as those targeting the 3′ end (Fig. 2b), indicating that the integrity of the aRNA amplified from both CTCs and microdissected FFPE PCa bone metastases was good. Having confirmed that the quality of the amplified aRNA was adequate, we analyzed all the CTC and BMBx samples included in the study using RT-PCR.

**Fig. 3:**
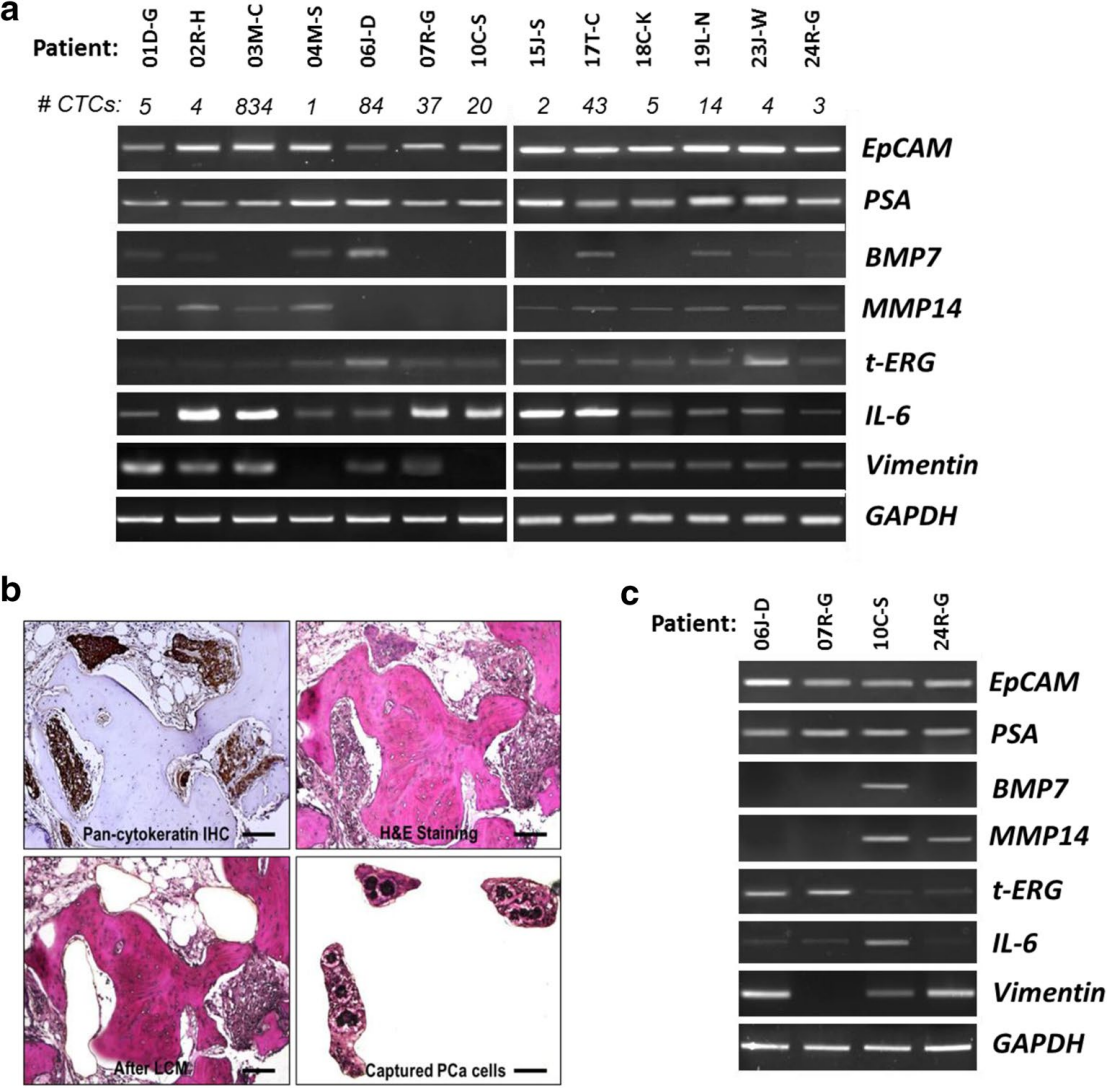
Gene profiles shown by amplified aRNA obtained from CTCs and PCa tissue microdissected from FFPE BMBx. **a** RT-PCR analysis of aRNA amplified from CTCs. **b** Representative section obtained from a FFPE BMBx of patient 24R-G, with PCa metastatic to bone, as confirmed by pan-CK immunostaining (*top left picture*). An adjacent section stained with H&E (*top right*) was used to microdissect (*bottom left*), and capture (*bottom right*) areas containing PCa cells only. Bar magnification, 200 μm. **c** RT-PCR analysis of aRNA amplified from PCa microdissected from FFPE BMBx from four mCRPC patients

Of 24 patients with bone scan/CT scan evidence of one or more skeletal metastases enrolled in the study, nine had no CTCs and therefore did not undergo bone biopsy. Table 1 summarizes the demographic, clinical, and tumor characteristics of the 15 patients that had one or more EpCAM-positive CTCs in their blood. One patient was eliminated from the study due to undetectable aRNA in CTCs even after two-rounds of amplification. In addition, another patient was excluded because his BMBx showed sarcomatoid tumor cells. Of the 13 patients remaining, a second blood sample was obtained and subjected to CTC enrichment using the CellSearch^®^ Profile kit for RNA extraction, and subsequent aRNA amplification. RT-PCR analysis of aRNA amplified from CTCs revealed that the gene expression varied from one patient to another. As expected, *EpCAM*, which codes for the protein used for CTC enrichment, was expressed in all cases. *PSA* and *IL*-*6* were also expressed by CTCs from all patients (Fig. 3a). Unexpectedly, the transcript for *Vimentin* was expressed in many of patients’ CTCs (Fig. 3a). Importantly, aRNA amplification from as little as one CTC (e.g., patient 04 M–S) was sufficient to perform gene analysis, consistent with our results with amplified aRNA obtained from five cultured PCa cells.

Of 13 patients with detectable CTCs, 7 (54 %) had cytokeratin-positive cells on immunohistochemical analysis of their BMBx. This yield is consistent with that reported in the literature [41, 42]. However, only four had a large enough area occupied by cancer cells for LCM and RNA collection. In those cases, we compared aRNA amplified from RNA extracted from ground flash frozen BMBx with that obtained from LCM FFPE sections of matching BMBx of the same patient. In the latter, areas exclusively containing PCa cells, as identified by H&E and confirmed by IHC for pan-CK, were UV laser microdissected from the bone specimens mounted on membrane glass slides, and captured with the help of a gentle IR laser, using the ArcturusXT™ LCM System (Fig. 3b). We observed that, although RNA yield was better in ground frozen bone core biopsies, the expression level of most of the genes specific to PCa was considerably diminished compared to amplified aRNA derived from PCa cells microdissected from FFPE BMBxs after equal number of PCR cycles and normalization to *GAPDH* (not shown). Conversely, genes commonly expressed by bone cells, such as *RANK* (receptor activator of nuclear factor κ-B), were highly expressed in aRNA derived from ground frozen BMBx, but minimally or not expressed in pure PCa cell populations acquired through LCM in FFPE BMBx (not shown). These results demonstrate that analysis of frozen bone samples is limited by contamination with normal bone cells.

Using at all times RNase-free instruments and technique, and aqueous reagents prepared in nuclease-free water, we were able to amplify good quality aRNA from the total RNA collected from the FFPE BMBxs (Fig. 2b). Since the aRNA yield obtained was good, we analyzed in BMBxs the same genes studied in CTCs (Fig. 3c). In order to explore whether or not there was concordance in presence or absence of gene expression as detected by conventional RT-PCR in CTCs and matched BMBxs, we lined up digitally the bands obtained for all the genes amplified in CTCs and BMBxs (same number of cycles) side by side (Additional file 1: Figure S1). We found that in 21 out of 28 comparisons, the presence or absence of detectable gene expression in CTCs and PCa cells microdissected from single bone metastasis of the same patients was concordant.

## Discussion

Precise analysis of human tissue is necessary for evaluation of gene expression. In this study, we wished to develop methods of gene expression analysis of CTCs and BMBxs in patients with mCRPC. We found that LCM, in conjunction with reliable methods of gene amplification, is an option for the specific isolation and molecular analysis of small number of PCa cells present in BMBxs. In recent years, LCM systems with IR and UV lasers combined with efficient software have been developed to isolate homogeneous cells precisely identified from heterogeneous tissues based on morphological criteria, and complemented by IHC phenotyping of the cell type of interest. In these LCM systems, the low-energy IR laser is fired to adhere spots within the selected tissue area to the PEN membrane of the glass slide, while the UV rapidly and precisely cuts out the zone defined to collect it in a collection cap [43]. In addition to RNA analysis, different types of molecular analyses can be performed on cells procured by LCM, including DNA and proteomic analyses [44, 45].

In this exploratory study with a small cohort of mCRPC patients, we demonstrated the feasibility of amplifying aRNA for gene analysis in limited numbers of PCa cells microdissected from FFPE BMBxs. We show that using a modified Eberwine’s procedure, adequate yields of RNA can be obtained for gene expression profiling. Our results obtained under tightly controlled conditions show good quality aRNA not only in freshly isolated CTCs, but also in aRNA amplified from FFPE BMBxs despite several studies having reported RNA degradation in formalin-fixed samples [46–48]. To avoid RNA degradation, we found that critical measures such as rapid processing of BMBxs after collection, preparation of all aqueous solutions using DEPC-treated water, and decontamination of all materials and surfaces with ready-to-use surfactants that remove RNA and RNases from lab equipment, are mandatory throughout the procedure. Previous studies have reported LCM and DNA sequencing from a frozen bone metastasis of a mCRPC patient [49], or gene expression analysis of total RNA directly obtained from snap-frozen bone marrow biopsies largely replaced by tumor [50] or frozen bone metastatic cores isolated at autopsy [12, 13, 51, 52]. However, in addition to our earliest analysis in a few PCa bone metastasis [53], the present study is the only report that, to the best of our knowledge, uses LCM and aRNA amplification for gene analysis of limited number of tumor cells microdissected from FFPE BMBx obtained from living mCRPC patients. In spite of better RNA recovery and quality ascribed to frozen tissue [43, 44, 54], we found that its use as homogenate might be inadequate for gene expression analysis of PCa bone micrometastasis, due to dilution of PCa-specific genes by other genes expressed predominantly by bone cells. In that sense, the use of LCM to identify and capture precise cells from FFPE BMBxs seems to be more accurate to study gene expression after aRNA amplification. Limitations of this approach include the yield of bone biopsies (~50 %) and the need for sufficient tissue (~9000 µm^2^) for LCM.

Besides the clinical utility of the CTC count to monitor treatment response and predict survival in mCRPC patients [11], enriched CTCs can also be used for molecular studies that may provide important clues to understand the biology behind metastatic dissemination of PCa. Analysis of CTCs may also lead to the discovery of novel predictive and prognostic biomarkers or demonstrate targets for therapy. Many groups have succeeded in genomic and transcriptomic profiling of CTCs in patients with PCa [29, 55–62]; however, to our knowledge, none of them have compared molecular features of CTCs with those of bone micrometastasis in mCRPC patients. Because significantly higher CTC numbers are detected in mCRPC patients with bone metastasis relative to those without bone metastatic lesions [38], CTCs in patients are likely to derive from bone metastatic deposits. Thus, we hypothesized that gene signatures of skeletal metastasis may be mirrored in CTCs in each mCRPC patient. Our study suggests some concordance in presence or absence of gene expression in CTCs and single bone lesions of the same mCRPC patients. An obvious limitation of our study is the small sample size and the biopsy-targeting of single bone lesions; future studies with more patients and perhaps more biopsy sites in individual patients may provide additional clarification.

Data collected from autopsy studies in men who died of mCRPC have revealed substantial heterogeneity among tumor cells in bone metastases within the same patient [63–65]. Therefore, we surmise that presence or absence of detectable gene expression in CTCs, rather than gene expression levels, may provide a more clinically relevant overview of the genomic landscape in each mCRPC patient. Detectable gene expression in CTCs may suggest a druggable target; however, this result may ultimately be unreliable due to heterogeneity of target gene expression among separate metastatic deposits within individual patients. Similarly, absence of target gene expression in CTCs may not rule out target gene expression in some metastatic deposits. Therefore, this exploratory analysis raises concerns about relying on CTCs to predict response to therapy.

In this study we used CellSearch^®^, an EpCAM-based platform utilized for capturing of epithelial tumor cells from peripheral blood of mCRPC patients. Like other CTC-enriching technologies that rely on the expression of epithelial markers, the CellSearch^®^ system does not have the capacity to isolate CTCs that might have gone through EMT. However, we found that the genes that code for the epithelial marker EpCAM and the mesenchymal marker vimentin were co-expressed in many of our patients’ CTC samples, suggesting a partial EMT. These results are in agreement with the findings of other groups that reported expression of EMT markers in EpCAM-positive CTCs [66–68]. We also found *Vimentin* to be expressed by most of the pure PCa tissues microdissected from BMBx. Our results are supported by IHC studies by Sethi et al. that show vimentin overexpression at the invasive front of bone metastases of PCa patients and E-cadherin within the center of the lesion [69], and those of Bryden et al. who reported lower E-cadherin expression in poorly differentiated bone metastases than in more differentiated ones [70]. The expression of mesenchymal markers in PCa metastatic to bone could be ascribed to osseous factors that offer a selective growth advantage for tumor cells with a mesenchymal phenotype, as opposed to other tissues (e.g., lung) that promote mesenchymal-to-epithelial transition in colonizing cancer cells [71, 72].

## Conclusions

To our knowledge, this exploratory study is the first to demonstrate the feasibility of simultaneous gene analysis in small numbers of CTCs and laser-captured, microdissected bone metastases in living mCRPC patients. Our data suggest that partial EMT might take place in bone metastatic PCa cells and in CTCs captured using EpCAM-based enrichment platforms. In this study, we found some concordance in gene expression profile of CTCs and PCa cells microdissected from single bone lesions of the same patients. This exploratory analysis raises concerns about relying on CTCs to predict response to therapy. Further investigations using a larger cohort of patients and new technologies capable to enrich the full spectrum of CTCs are needed to define more precisely the genomic landscaping of mCRPC through the use of these “liquid biopsies”.

## Additional file

10.1186/s12967-016-0829-5 Side by side comparison of gene expression (detectable/non-detectable) shown by aRNA from CTCs and BMBx after reverse transcription and PCR with same number of cycles.
