# Supplementary figures and images for: Gene expression analysis of bone metastasis and circulating tumor cells from metastatic castrate-resistant prostate cancer patients
Source: J Transl Med. 2016 Mar 15;14:72. doi: 10.1186/s12967-016-0829-5 (PMC4791970; doi:10.1186/s12967-016-0829-5)

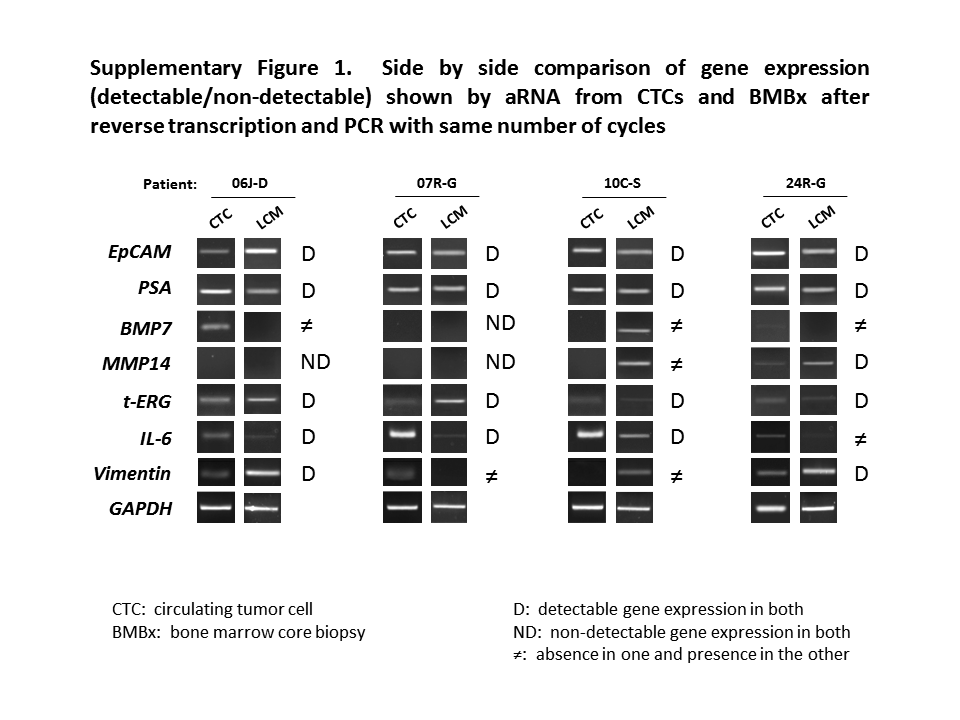

Supplement: Supplementary file 1 — 10.1186/s12967-016-0829-5 Side by side comparison of gene expression (detectable/non-detectable) shown by aRNA from CTCs and BMBx after reverse transcription and PCR with same number of cycles. [file 12967_2016_829_MOESM1_ESM.tif]
